# Assessing Evidence for a Common Function of Delay in Causal Learning and Reward Discounting

**DOI:** 10.3389/fpsyg.2012.00460

**Published:** 2012-11-15

**Authors:** W. James Greville, Marc J. Buehner

**Affiliations:** ^1^College of Medicine, Swansea UniversitySwansea, UK; ^2^Department of Psychology, Swansea UniversitySwansea, UK; ^3^School of Psychology, Cardiff UniversityCardiff, UK

**Keywords:** causal learning, delay discounting, reinforcement delay, subjective reward value, utility

## Abstract

Time occupies a central role in both the induction of causal relationships and determining the subjective value of rewards. Delays devalue rewards and also impair learning of relationships between events. The mathematical relation between the time until a delayed reward and its present value has been characterized as a hyperbola-like function, and increasing delays of reinforcement tend to elicit judgments or response rates that similarly show a negatively accelerated decay pattern. Furthermore, neurological research implicates both the hippocampus and prefrontal cortex in both these processes. Since both processes are broadly concerned with the concepts of reward, value, and time, involve a similar functional form, and have been identified as involving the same specific brain regions, it seems tempting to assume that the two processes are underpinned by the same cognitive or neural mechanisms. We set out to determine experimentally whether a common cognitive mechanism underlies these processes, by contrasting individual performances on causal judgment and delay discounting tasks. Results from each task corresponded with previous findings in the literature, but no relation was found between the two tasks. The task was replicated and extended by including two further measures, the Barrett Impulsiveness Scale (BIS), and a causal attribution task. Performance on this latter task was correlated with results on the causal judgment task, and also with the non-planning component of the BIS, but the results from the delay discounting task was not correlated with either causal learning task nor the BIS. Implications for current theories of learning are considered.

## Introduction

The role of time is central to learning and behavioral processes. The precise temporal arrangements of when we perform actions, when the consequences of those action manifest, and when other events occur alongside these, can have a profound influence on the way in which such events are interpreted. Researchers in fields as diverse as neurology, computer science, and psychotherapy have long been interested in the ways in which our behavior is sensitive to time, and which psychological processes and underlying neurological structures govern such activity.

Reinforcers or rewards are stimuli that elicit a change in the behavior of an organism. Though virtually any stimulus has the potential to reinforce behavior, the typical conception of a reward is that which has a particular motivational significance or adaptive value to the organism, such as food. Rewards can in many cases be quantified (for instance, the volume of food received) and in this regard have an objective value. As one might expect, animals exhibit preference for larger rewards over smaller rewards. However, depending on the current situation (such as the animal’s level of deprivation) the reward may also have a subjective value that differs from its objective magnitude. A factor of crucial importance in determining the subjective value is the time when a reward is received. Naturally, immediate rewards are preferred to delayed rewards, when the rewards are of equal magnitude; however, numerous studies have demonstrated that in certain cases, animals will choose a smaller, immediate reward over a larger, delayed reward. If we assume that the animal always selects the reward which it perceives has the greater value, then we may conclude that the subjective value of a reward declines with delay. Delays of reinforcement thus result in the objective value of the reward being discounted, hence the term delay discounting is used to describe this process.

The rate at which rewards are discounted as the delay increases varies between individuals. Those for whom the value of rewards declines steeply with delay are often identified as impulsive, since their routine preference for rapid reinforcement implies an inability to delay gratification in order to receive a larger reward. Studies have found differences in the rate of discounting between different age groups (Green et al., 1994, 1999) and cultures (Du et al., 2002). However, the general shape of the discounting function tends to be the same across individuals. A considerable effort has been made by a number of researchers (Mazur, 1987; Rachlin et al., 1991) to identify the mathematical relation that best describes the relationship between the delay until a reward is received and its subjective value. Initial work found that both an exponential decay function, *V *= *Ae*^−*kD*^, and simple hyperbola, *V* = *A*/(1 + *kD*), provided reasonable fits to discounting data, where *V* is the current subjective value, *A* is the nominal amount of the reward, *D* is the delay to reward, and *k* is a free parameter, representing the steepness of the discounting function. Myerson and Green (1995) concluded that the function most closely mapping how subjective value changes with delay is a hyperbola-like function with the addition of a scaling parameter: *V* = *A*/(1 + *kD*)*^s^*, where the exponent *s* represents the non-linear scaling of amount and time; in other words, *s* has the effect of causing the curve to decline more slowly at long delays.

Obtaining a reliable measure of discounting can be problematic because of the lack of consensus over the mathematical function best suited to fit discounting data, and the difficulty involved in estimating the parameter *k*. To address this, Myerson et al. (2001), proposed the novel measure of obtaining the area under the curve (AUC) of the empirical discounting function. For this to be calculated, the points on a plot of the function are connected using straight lines and the area below the line can then be obtained using a fairly simple calculation. Further details of this procedure are provided in the Section “Materials and Methods” of this paper. AUC provides a simple, parameter-free measure of discounting that is not tied to a specific theoretical framework. It has the advantage of being applicable to individual or group data, and furthermore allows for direct comparison of discounting rates, whether between individuals or across tasks involving different amounts of reward or delay.

Delays also play a central role in conditioning, appearing to interfere with the acquisition process, with behavior taking longer to establish (Wolfe, 1921; Solomon and Groccia-Ellison, 1996) and being diminished either in magnitude or in rate (Williams, 1976; Sizemore and Lattal, 1978). Plots of the decline in response rate against time reveal similarly negatively accelerated functions as for delay discounting. Chung (1965) found in a signaled-delayed-reinforcement task that pigeons’ response frequencies declined exponentially as a function of the delay interval. Other work (Herrnstein, 1970; Mazur, 1984) suggests that hyperbolic functions more accurately describe the trends in response data with delays. As with discounting, there is a lack of consensus regarding the precise shape of the function describing how response rates decline with delay. However, it is generally agreed that the relationship may be broadly described as a negatively accelerated decay function. A commonality between the process of temporal discounting and associative learning may thus be identified, raising the possibility that the two processes may have a shared cognitive basis. Indeed, some researchers (Dickinson et al., 1984; Dickinson, 2001) posit that many aspects of what is commonly referred to as higher-level human learning and cognition are fundamentally governed by simple associative mechanisms. Others adopt the viewpoint that processes such as induction and reasoning are based on more complex computational (e.g., Cheng, 1997) or symbol-manipulating (e.g., Holyoak and Hummel, 2000) architectures. However, such processes are still subject to the effects of time, as shall now be discussed.

Causal inference is the process by which we come to learn that an event has the capacity to produce or otherwise influence another event. Acquiring the knowledge that one event leads to another is fundamental not only to understand why events occur, but to direct our own behavior to intervene on the world and bring about desired outcomes. Causal inference is referred to as such because we cannot directly perceive a causal relation, and causality must therefore be inferred from the observable streams of evidence that are available to us. Hume (1888) identified three cues to causality: temporal precedence, contingency, and contiguity. To elaborate, causes must precede their effects, be followed by their effects with sufficient regularity, and be closely coupled in time (and space) with those effects.

Time is therefore a bedrock of causal induction according to the Humean doctrine, with contiguity essential for learning to take place. Initial research, approaching causal induction from an associative learning perspective, indeed supported this view. Shanks et al. (1989) found that in judging contingency between pressing a button and a triangle illuminating on a computer screen, human participants were unable to distinguish conditions involving delays of 4 s or greater from non-contingent conditions where the probability of the outcome was just as likely in the presence and absence of the cause. Such findings appear puzzling since both humans and animals demonstrate the ability in a variety of tasks to learn delayed causal relations. Recent research has demonstrated that there are a number of factors mitigating the effects delay such as prior knowledge or previous experience and resultant expectation (Einhorn and Hogarth, 1986; Buehner and May, 2003, 2004), awareness of causal mechanism (Buehner and McGregor, 2006), or structural information in the environment (Greville et al., 2010). Nevertheless, it is generally recognized that delays create difficulties for causal induction and that all other things being equal, a reasoner is more easily able to identify contiguous causal relations than those involving a delay. Studies such as those of Shanks et al. (1989; see also Shanks and Dickinson, 1991) show that causal ratings do tend to follow a pattern of decline with time that is similar to the decline of response rates in reinforcement learning with animals, with a sharp fall in ratings from immediate to delayed causal relations, with the steepness of the curve easing and flattening as delays extend.

Thus, there is a common effect of delays in associative learning, causal induction, and delay discounting. While it may be a stretch to posit that they are all essentially the same cognitive process, it seems reasonable enough to suggest that the way by which delays are recognized, interpreted, and represented may involve a common mechanism that forms a crucial part of all these processes. The effects of delay may vary from person to person, and from task to task, but it seems plausible that if delays are interpreted via a stable underlying process, then there should be some perceptible pattern in the way in which delays generally affect the behavior of an individual. Having then identified a common cognitive contribution of delay across learning processes, we now turn to consider evidence of how delays may be represented from a neurobiological perspective, and whether these processes all involve a common region of the brain that may be the site of temporal processing.

While the effects of reinforcement delay on behavior have been extensively studied, the neurobiological basis of such effects has received comparatively less attention (Evenden, 1999). However, it is well-established that the hippocampus plays an important role generally in learning and memory. Solomon et al. (1986) demonstrated that an intact hippocampus is required for trace conditioning but not delay conditioning in rabbits^1^. Beylin et al. (2001) demonstrated that hippocampal lesions in rats also impair delay conditioning when a longer inter-stimulus interval is used. This suggests that the hippocampus plays a role in the formation of associations between temporally discontiguous stimuli.

Bangasser et al. (2006) postulated that the hippocampus was responsible for forming an active representation of the CS that could then be associated with the US. Using a novel “contiguous trace conditioning” (CTC) paradigm, where the standard trace conditioning preparation was modified by representing the CS simultaneously with the US following the trace interval, Bangasser et al. demonstrated that hippocampal-lesioned rats could successfully condition with this procedure. Related findings by Woodruff-Pak (1993) concerning the patient HM, were interpreted by Bangasser et al. as evidence that existing association between the stimuli (as a result of previously experienced temporal contiguity) is required for trace conditioning with hippocampal damage. They speculate that the function of the hippocampus in conditioning is to bind stimuli that do not occur together in time.

Cheung and Cardinal (2005), however, obtained results that appear to directly oppose those of the above studies. In an action-outcome (i.e., instrumental) learning task, hippocampal-lesioned animals actually became better at learning (relative to shams) as the delay between action and outcome increased. Cheung and Cardinal explain this effect by suggesting that normal hippocampal function promotes the formation of context-outcome associations. In instrumental conditioning then, context-outcome associations compete with and thus hinder learning of response-outcome associations, so a disruption of contextual processing via hippocampal lesion will improve learning with delayed outcomes. Meanwhile during classical conditioning the CS may be considered part of the context and thus the reverse effect is obtained. In yet a further twist, Cheung and Cardinal found that the same lesioned animals were also poorer at choosing a delayed larger reward over an immediate smaller reward – despite their apparently superior ability at learning the predictive relationship between action and outcome when delays were involved. In other words, lesioned animals made more impulsive choices relative to shams.

Similar findings were obtained by McHugh et al. (2008) using a T-maze task. Rats chose between the two goal arms of a T-maze, one containing an immediately available small reward, the other containing a larger reward that was only accessible after a delay. Hippocampal lesions reduced choice of the larger delayed reward in favor of the smaller immediately available reward. McHugh et al. advanced the argument that the hippocampus assists normal temporal processing by acting as intermediate memory store that allows animals to associate temporally discontiguous events, and that insertion of a delay into tasks will result in abnormal performance in animals with hippocampal damage.

In summary then, the hippocampus has been implicated both in the process of choice between delayed rewards, and in conditioning processes. While the empirical evidence does not precisely elucidate the role of the hippocampus, there is clear indication that it is involved in processing temporal and contextual information. Specifically, the temporal processing that appears to be a necessity for trace conditioning or the delay of gratification to receive a larger reward is hippocampal-dependent. Thus, it seems logical to query whether both processes appeal to the same neural mechanism, and thus whether there may be a common process by which delayed rewards lose their subjective value and associative strength or impression of causality declines with delay.

Having reviewed a number of behavioral and biological findings, there seems to be mounting evidence that the processes of reinforcement learning and intertemporal choice behavior may well share a common foundation. We investigated the behavioral evidence that could lend credence to a hypothesis of shared function. More specifically, we pursued an individual differences approach, where we related an individual’s performance in a standard causal learning task to their degree of temporal discounting to ascertain whether the two are correlated. It seems that whatever the outcome, there may be important implications for our understanding of timing behavior, in particular with regard to providing a unified theory of learning.

## Experiment 1

Our goal for the first empirical study was to contrast behavior at the individual level on two well-established paradigms. Each participant completed two studies, a causal judgment task and a delay discounting procedure. It is important here to note that that the former, although an instrumental task, was evaluative rather than performance-based. In a typical instrumental performance task, the outcome has some appetitive value; such as a food reinforcer in animal reinforcement learning, or scoring points in a simple game context (Shanks and Dickinson, 1991) for tasks with human participants. Such tasks can often be complicated by the payoff matrix – that is the benefit of the outcome compared to the cost of responding. A causal judgment task meanwhile is free from such complications; the outcome is not assigned a particular value and the participant is given no motivation to try and make the outcome occur as much as possible. Rather, participants are simply given time to investigate and evaluate the causal relationship between response and outcome, selecting their own response strategy and providing a declarative judgment of contingency. Employing such a task thus enabled us to probe causal learning in an uncompromised manner.

### Materials and methods

#### Participants

Ninety-one undergraduates from Cardiff University, 28 males and 63 females, with an average age of 20 years, volunteered to participate as part of a practical class. Participants did not receive any payment for participation. Due to computer malfunction, data for two participants was lost for the delay discounting task.

#### Design

The experiment consisted of two components, a causal judgment task, and a delay discounting task. The causal judgment task manipulated the independent variables contingency (or more accurately *P*(e|c), the probability of an outcome given a response), and delay between response and outcome. Two levels of contingency (0.50 and 0.75) were factorially combined with three levels of delay (0, 2, and 5 s) to produce six experimental conditions, each of 120 s duration, in a 2 × 3 within-subjects design. With condition order counterbalanced across participants. The dependent measure was the causal rating (0–100) provided by participants at the end of each condition.

The delay discounting task combined two levels of the factor reward (£200 and £10,000) with seven levels of the factor delay (1 month, 3 months, 9 months, 2, 5, 10, and 20 years) in a 2 × 7 within-subjects design. The dependent measure in each case was the point of subject equivalence (see below for how this was determined). Taken together, the points of subjective equivalence at each level of delay (for a given reward amount) yielded the AUC (again see below) which was the main dependent measure we used.

#### Apparatus and procedure

The two tasks were programmed using Python version 2.4.1 for the causal learning task and E-Prime version 2.0 from Psychology Software Tools for the delay discounting task. Each participant used a PC running Windows XP with a 19” LCD widescreen display, using a standard mouse and keyboard to input responses. The experiment was conducted in a small computer lab, with participants seated at individual workstations which were screened off from each other.

The causal judgment task was closely modeled on Shanks et al.’s (1989) study. For each condition, an outline of a triangle was displayed on the computer screen and beneath this a button which could be pressed by clicking on it with the mouse. Participants engaged in a free-operant procedure (FOP), where they were permitted to respond at any point and as often as they wished, with each response subjected to the reinforcement schedule. Every press therefore had the specified probability (either 0.5 or 0.75) of generating an outcome. If an outcome was scheduled, the triangle illuminated (the gray background became red and a “glow” effect appeared around the outline) for 250 ms following the programmed delay (either 0 s, i.e., immediately, 2 or 5 s). For all conditions, the triangle also illuminated unprompted once every 10 s period at a random point within that 10 s period – in other words, the first such background effect could occur at any time between 0 and 10 s, the second between 10 and 20 s, and so on. These random background effects were included to add a degree of uncertainty as to whether a given outcome was indeed generated by a response made by the participant or due to unseen alternate causes, thus making the task non-trivial. Each condition lasted for 2 min, at the end of which participants were asked “how effective is pressing the button at causing the triangle to light up?” and instructed to provide a rating from 0 to 100.

The delay discounting task was essentially a replication of Du et al.’s (2002) experiment. Combination of the factors amount and delay provided 14 different conditions, presented in a different random order for each participant. On-screen instructions and three practice trials were presented prior to beginning the experiment. It was made clear to participants that the amounts of money were hypothetical and they would not receive any real money for participating in the study. Each condition comprised seven choices or trials. For a given trial, participants were presented with two boxes, one containing the smaller, sooner (SS) reward and one containing the larger, later (LL) reward, and required to indicate which of these rewards they would prefer to receive. The left-to-right placement of the two rewards was randomized from trial to trial. Participants pressed Q or P on the keyboard to select the left or right reward respectively.

The value of the LL reward was always fixed at the specified amount of either £200 or £10,000, and the time until its receipt was one of the seven delays. The SS reward could be obtained “now,” and its value changed from one choice to the next. For the first choice within each condition, the value of the immediate reward was half that of the delayed reward (e.g., £5000 now vs. £10,000 in 10 years). If the SS reward was chosen, its value was decreased on the subsequent choice; if the LL was preferred, the value of the SS was increased. The amount by which the SS was adjusted was half of the difference between the two rewards (i.e., £2500 in the above example). Thus if the participant chose £5000 now, the next choice would be between £2500 now and £10,000 in 10 years; if they chose the £10,000, the next choice would be between £7500 and £10,000. The amount of the adjustment was rounded to the nearest integer. This “titration” procedure was designed to converge on the subjective value of the LL reward. The subjective value was calculated as the mean of the last immediate reward that had been chosen and the last immediate reward that had been rejected.

To calculate the AUC, we normalized delay and subjective value by expressing each delay as a proportion of the maximum delay (20 years, i.e., 240 months), and each subjective value as a proportion of the nominal amount (i.e., £200 or £10,000). These proportions were then used as *x* and *y* coordinates respectively to graph each individual’s discounting function. Connecting the individual data points using straight lines effectively divides the graph into a series of trapezoids, with the sum of the areas of all the trapezoids providing the total AUC. These areas can be calculated without actually constructing the graph, by using the simple formula: (*x*_2_−*x*_1_) × [(*y*_1_ + *y*_2_)/2] for each trapezoid, where *x*_1_ and *x*_2_ are successive delays and *y*_1_ and *y*_2_ are the corresponding subjective values. For the first trapezoid, the values of *x*_1_ and *y*_1_ are 0 and 1 respectively. Since the *x* and *y* values are proportions, the maximum AUC is 1 (i.e., no discounting) with smaller values representing steeper discounting.

### Results

#### Causal judgment task

**Figure 1:**
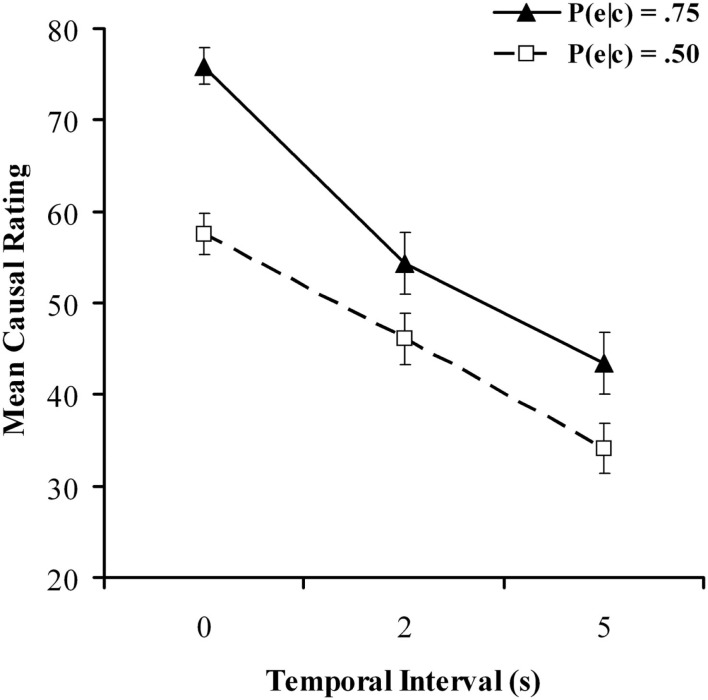
**Mean causal ratings for all conditions in the causal judgment task as a function of temporal delay for the causal judgment task in Experiment 1**. Filled and unfilled symbols refer to *P*(e|c) values of 0.75 and 0.50 respectively.

Figure 1 shows mean causal ratings for all six conditions in the causal judgment task. As expected, ratings were considerably higher at *P*(e|c) = 0.75 than at *P*(e|c) = 0.50. Also in accordance with our expectations, ratings declined as the delay between cause and effect increased. A 2 × 3 repeated-measures ANOVA confirmed significant main effects effect of contingency, *F*(1, 90) = 39.69, *p *< 0.001, *MSE* = 470, $\eta_{p}^{2}\;=\;0.306$ and delay, *F*(2, 180) = 52.61, *p *< 0.001, *MSE* = 640, $\eta_{p}^{2}\;=\;0.369$ as well as a significant interaction between contingency and delay, *F*(2, 180) = 4.12, *p *< 0.05, *MSE* = 437, $\eta_{p}^{2}\;=\;0.044$ Closer inspection of Figure 1 reveals that the difference between judgments at *P*(e|c) = 0.75 and *P*(e|c) = 0.50 was noticeably greater with 0 s delay than with 2 or 5 s, which is likely the driving force behind the significant interaction. The main effects are in accordance with several previous findings in the literature; a similar interaction meanwhile was also found by Shanks et al. (1989) when contrasting experimental conditions with *P*(e|c) = 0.75 against control conditions with *P*(e|c) = 0. Although our study instead used values of 0.75 and 0.5, this finding is broadly consistent with the idea that delays make it harder to recognize and differentiate between objective contingencies and that contingency and contiguity act in concert to influence perception of causality (Greville and Buehner, 2007).

**Figure 2:**
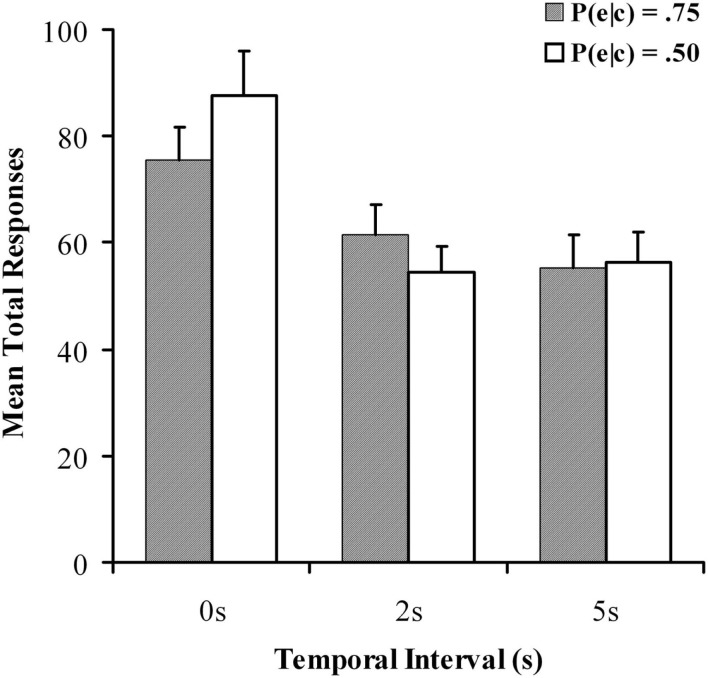
**Mean total responses for all conditions in the causal judgment task as a function of temporal delay for the causal judgment task in Experiment 1**. Filled and unfilled bars refer to *P*(e|c) values of 0.75 and 0.50 respectively.

Mean response rates per minute are reported in Figure 2, and a 2 × 3 within-subjects ANOVA was again used to examine differences between conditions. An analysis of response rates found no significant effect of contingency, *F*(1, 90) = 0.483, *p *= 0.489, *MSE* = 1118; however there was a main effect of delay on response rates, *F*(2, 180) = 28.582, *p *< 0.001, *MSE* = 1305, $\eta_{p}^{2}\;=\;0.241$ with fewer responses emitted during the delayed conditions, in line with existing findings (Reed, 1999; Buehner and May, 2003). The implication is that participants withhold further responding until the consequences of their actions are revealed, leading to fewer responses with longer delays. There was also a significant contingency × delay interaction, *F*(2, 180) = 3.973, *p *< 0.05, *MSE* = 10597, $\eta_{p}^{2}\;=\;0.042$ response rate with 0 s delay was significantly greater at *P*(e|c) of 0.5 than at 0.75. These extra responses could account for the interaction observed in the causal ratings, in line with a negative outcome density effect. In an appetitively neutral task such as this however, response rate may not indicate much about causal beliefs and it is the causal judgment that should be focused on as the critical measure.

#### Delay discounting task

**Figure 3:**
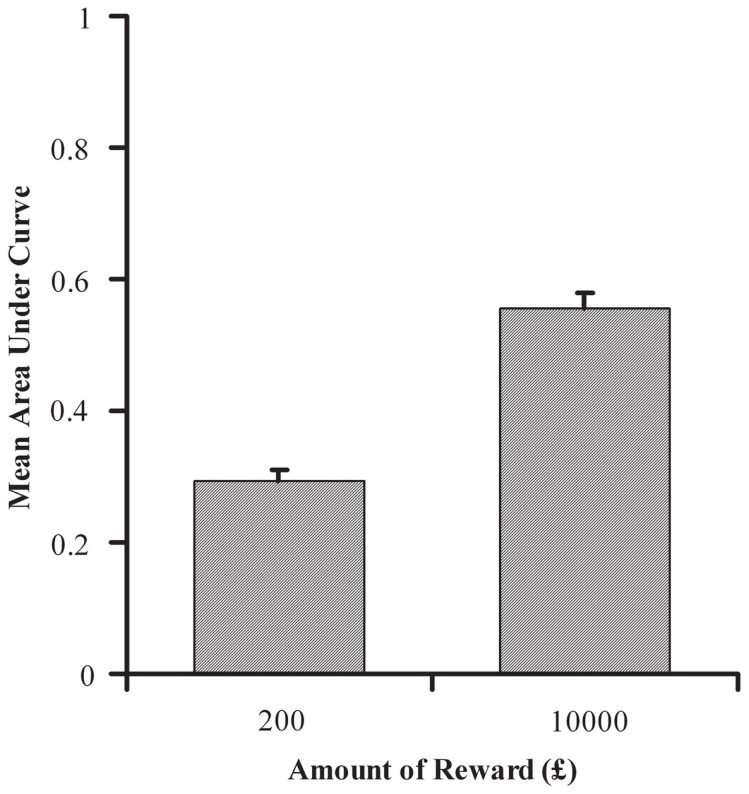
**Mean area under the curve (AUC) as a function of delayed reward amount for the delay discounting task in Experiment 1**. AUC is calculated from participants’ point of indifference at combinations of delay extent and value of immediate reward.

Using the points of subjective equivalence, the AUC was calculated at each amount of reward for all individual participants, as specified in the Section “Materials and Methods”. Mean AUC for £200 and £10,000 are shown in Figure 3. AUC was significantly greater with delayed rewards of £10,000 than £200, *t*(88) = 12.138, *p *< 0.001, indicating that discounting was less severe with the larger reward and thus replicating established findings (e.g., Green et al., 1994). Although individuals tended to discount smaller amounts more steeply, they were consistent in the manner of their discounting across reward amounts, with a strong positive correlation between an individual participant’s AUC at £200 and £10,000, *r* = 0.541, *n* = 89, *p* < 0.001. This supports the idea that individual discounting functions at different reward amounts differ by a scaling factor, rather than by any qualitative difference in function shape.

#### Cross-task comparisons

We created a novel metric for each participant that allowed us to relate their performance on the causal judgment task to their individual level of delay discounting task. More specifically, we represented the manner in which an individual’s perception of causality declined with delay in a fashion analogous to AUC: we expressed each rating and each delay as a proportion of the maximum (100 and 5 s respectively). This allowed calculation of the AUC in the same manner as described for the delay discounting task, separately for judgments at *P*(e|c) = 0.75 and 0.50 as for £200 and £10,000. Henceforth we distinguish between the two measures using the terms AUC_c_ (for the causal judgment task) and AUC_d_ (for the discounting task).

AUC_c_ was significantly greater at *P*(e|c) of 0.75 than 0.5, indicative of the higher ratings attracted by the stronger contingency. There was however a strong significant positive correlation between individuals’ AUC_c_ at *P*(e|c) of 0.75 and that at 0.50, *P*(89) = 0.546, *p* < 0.001, much like that between AUC at £200 and £10,000 in the discounting task. Once again this demonstrates that delays affected individuals’ perception in a consistent manner, with causal judgments being devalued similarly within individuals across both levels of contingency. Males evaluated delayed causal relations more favorably than did female participants, both at *P*(e|c) = 0.75 (mean AUC_c_ were *M* = 0.60 and *M* = 0.53 for males and females respectively) and *P*(e|c) = 0.50 (*M* = 0.49 and *M* = 0.43), yet discounted delays slightly more steeply than females at both £200 (*M* = 0.28 and *M* = 0.30) and £10,000 (*M* = 0.53 and *M* = 0.56). However, none of these differences reached statistical significance (all *p*s > 0.1). The remainder of the analysis therefore collapses across gender.

**Figure 4:**
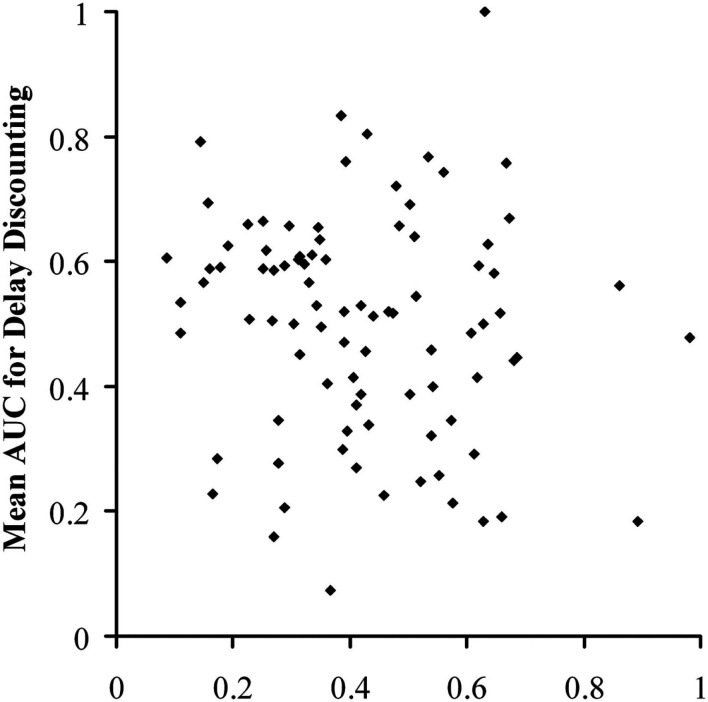
**Scatter plot of area under the curve for the discounting task against mean area under the curve for the causal judgment task for individual participants in Experiment 1**.

Since representing the delay-induced decline in causal judgments is not an established standard, we also calculated the ratios of the delayed to the immediate scores; specifically, ratings at 2 s over ratings at 0 s and ratings at 5 s over ratings at 0 s, for both *P*(e|c) = 0.75 and 0.50. This gave four individual ratios, plus a mean ratio across levels of contingency. This provided several bases of comparison to the AUC_d_ from the discounting task. Figure 4 shows a plot of all individual participants’ mean AUC_d_ against their mean AUC_c_. There was no correlation between these two scores, *r* = −0.114, *n* = 89, *p* = 0.289, nor was there a correlation between mean AUC_d_ and mean ratio of delayed to immediate judgments, *r* = 0.026, *n* = 89, *p* = 0.809. There was likewise no correlation between ratio and AUC_d_ for any of the possible eight comparisons between the AUC_d_ at the two nominal values and the four ratios (all *p*s > 0.2).

We set out to examine the relationship between delay discounting and delay-impaired causal judgment, aiming to identify whether individuals devalue delayed rewards in the same manner as they appraise delayed causal relations. Overall, our results replicate several well-established findings in the literature: both contingency and contiguity substantially impact perceived causality, with causal judgments increasing in line with the proportion of responses generating outcomes, and declining with response-outcome delay (Shanks et al., 1989); and larger rewards being discounted less steeply with delay than smaller rewards (Raineri and Rachlin, 1993). We also found good consistency at the individual level both between discounting functions at different amounts, as well as coherence between trends of delay-induced decline at different contingencies. We can thus have confidence both in the reliability of our paradigms and the measures adopted. However, of principle interest was whether a relationship existed between individual discounting function and evaluations of delayed causal relations. No such correlations were found with any of the comparisons we applied. The implication therefore is that the effect of a delay on an individual’s perception of causality is not related to the rate at which a reward loses its subjective value with delay. An extension of such a conclusion is that the discounting of a delayed reward is not based on an inability to identify or recognize a causal agent or mechanism that may be responsible for generating this delayed reward.

## Experiment 2

The failure to obtain a significant correlation in the first experiment is a result that is somewhat difficult to interpret. This could be taken as evidence that causal learning and choosing between concurrently available rewards are distinct processes, and do not share a common temporal processing mechanism. However, such a contention comes from the unenviable position of arguing from the null. Further investigation is therefore required. In the first instance, it seems most prudent to attempt to replicate these two studies and see if the same effect (or rather lack thereof) persists. At the same time, other measures can be introduced which may provide additional insight as to whether causal learning and discounting processes are in some way allied.

The following experiment then reprised effectively the same causal judgment and delay discounting tasks of the previous experiment (though both were streamlined as described in the Section “Materials and Methods”). In addition, two further tests were administered to participants. The first was a causal attribution task, in which participants had to select the true cause (or most likely cause) from three concurrently available causal candidates (further details will follow in the Section “Materials and Methods”). Performance in this task was used to compute a single metric that could be contrasted with the AUCs obtained from the causal judgment and discounting tasks. The second was version 11 of the Barrett Impulsivity Scale (BIS-11; Patton et al., 1995), a popular measure for assessing impulsive personality traits. Measures from all four tasks were then compared across participants.

### Materials and methods

#### Participants

A total of 71 participants with a mean age of 20 years took part in the study (29 males, 41 females, with one participant declining to disclose gender). Of these 71 participants, five failed to complete the causal judgment task, with one of these five also failing to complete the delay discounting task. One additional participant failed to complete the BIS-11, while one further participant failed to complete the causal attribution task. This gave a total sample sizes of 66, 70, 70, and 70 for each of the individual tasks respectively.

#### Design

The general format of both the causal judgment and the delay discounting tasks remained identical to that in the first experiment. However, given the additional tasks being included, it was decided to streamline both tasks in order to reduce the demands on participants. For the causal judgment task, we removed the second level of *P*(*e*|*c*), 0.5, making it a single-factor task with the same three levels of delay (0, 2 and 5 s), and *P*(*e*|*c*) set at 0.75 for each of these three condition. For the delay discounting task, the value of the LL reward was fixed at £200, with the additional value of £10000 dispensed with. As a result, the delay discounting task no longer remained as an experimental study in itself (since there were no independent factors) but instead contributed a single measure, AUC_d_, for later comparison with other tasks. Both AUC_c_ and AUC_d_ were calculated in exactly the same manner as for the previous experiment, however as a result of streamlining, rather than there being two values for AUC_c_ (at *P*(e|c) = 0.75 and *P*(e|c) = 0.5) and AUC_d_ (at LL = £200 and LL = £10000), there were only single values of each (at *P*(e|c) = 0.75 and LL = £200 respectively).

For the causal attribution task, the independent variable was the interval separating cause and effect. The dependent measures was whether the participant selected the true cause (accuracy) and the time taken for them to make their selection (response time). By taking mean accuracy and response times over all three conditions, we have two single measures which can be contrasted with AUCs for the initial two tasks and scores from the BIS-11.

The BIS-11 provides a total score with a possible range of 30–120, with higher scores indicating greater impulsiveness. The scale can be further subdivided into three second order factors, cognitive, motor, and non-planning, which can additionally be included in our cross-task comparisons.

#### Apparatus and procedure

All four tasks were completed in the same computer laboratory using the same equipment as for Experiment 1. Both the new causal attribution task and the administration of the BIS-11 were programmed using Python.

The new causal decision making task was adapted from Young and Nguyen (2009). They used a first-person-shooter (FPS) video game in which the participants’ task was to protect buildings that were being shot at by groups of three attackers. In each case, one attacker was an enemy and was firing explosive projectiles (the true cause, or target) while the other two were “friendlies” and firing duds (the foils). Participants could therefore protect the building by destroying the attacker that was causing the explosions. The key independent variable in Young and Nguyen’s experiment was the temporal interval between the true cause and the effect. Essentially then, the task can be summarized as deciding which of three candidate causes was producing an effect (explosions) by observing the timing of when each attacker fired its weapon and when the explosion occurred at the building.

In the causal attribution task for the current paper, we transferred the essential features of Young and Nguyen’s task from a 3D virtual environment to a simple experimental protocol using simple 2D stimuli, more closely resembling standard contingency judgment problems. Participants were presented with a triangle in the upper portion of the screen and below this was situated a row of three buttons. Alongside each button was a pointing hand, which would periodically press its adjacent button, constituting an instance of a candidate cause. The triangle illuminated contingent upon one of the buttons being pressed, with the other two buttons being foils. Buttons were labeled 1, 2, and 3 from left-to-right, and the position of the true cause on each condition was randomized on each condition. In our version of the task, participants were given three conditions in which the interval between the true cause and its effect was either 0, 2, or 5 s.

In governing stimulus delivery, an underlying trial structure was used in the same manner as for Young and Nguyen’s experiments, with the timeline divided into 4 s segments. This trial structure was not explicitly signaled to participants, and trials ran seamlessly from one into the next with each trial beginning immediately following the previous trial with no inter-trial interval. All the candidate causes (button presses) occurred during the first 3 s of each 4 s trial, randomly distributed within this 3 s. The effect then followed its true cause with the specified delay. The foils had no effect over the triangle. Trials continued until participants made their choice of which of the three candidates they felt was the true cause of the triangle lighting up.

The fourth and final task administered to participants was the BIS-11, a well known metric for assessing impulsiveness. The BIS-11 program presented all the items simultaneously on the same page. Participants clicked on labeled buttons to indicate the extent to which they agreed with each statement, and pressed a “submit” button to record these choices and calculate their score. All participants were administered all four tasks in the same fixed order, which was as follows: causal judgment, causal attribution, delay discounting, BIS-11.

### Results

#### Causal judgment task

**Figure 5:**
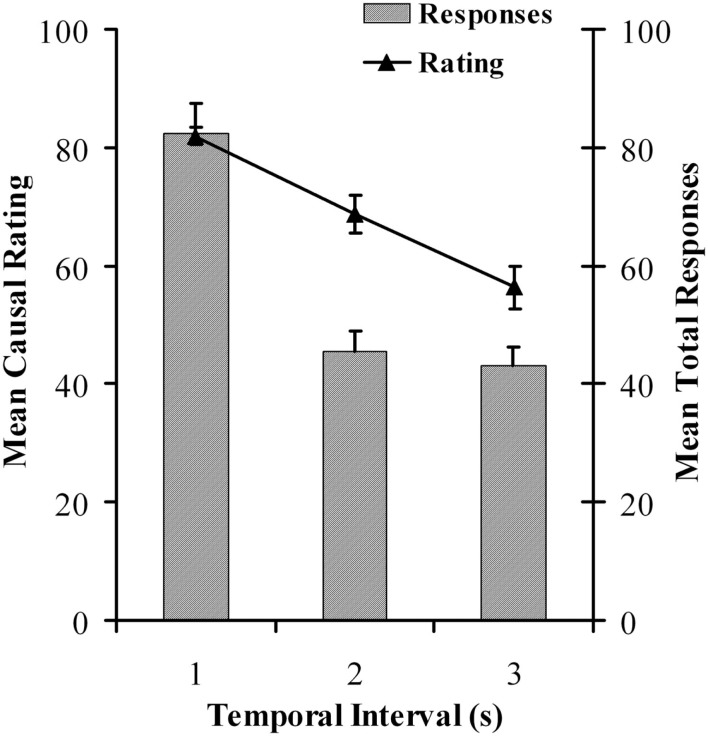
**Mean causal ratings and mean total response rates as a function of temporal delay for the causal judgment task in Experiment 2**.

Results for the causal learning task mirrored those of the previous experiment. Both causal ratings, *F*(1, 130) = 23.477, *p* < 0.0005, *MSE* = 458.747, $\eta_{p}^{2}\;=\;0.265$ and overall response rates, *F*(1, 130) = 42.790, *p* < 0.0005, *MSE* = 751.475, $\eta_{p}^{2}\;=\;0.397$ declined as a function of increasing cause-effect delay. The results are summarized in Figure 5.

#### Causal attribution task

**Figure 6:**
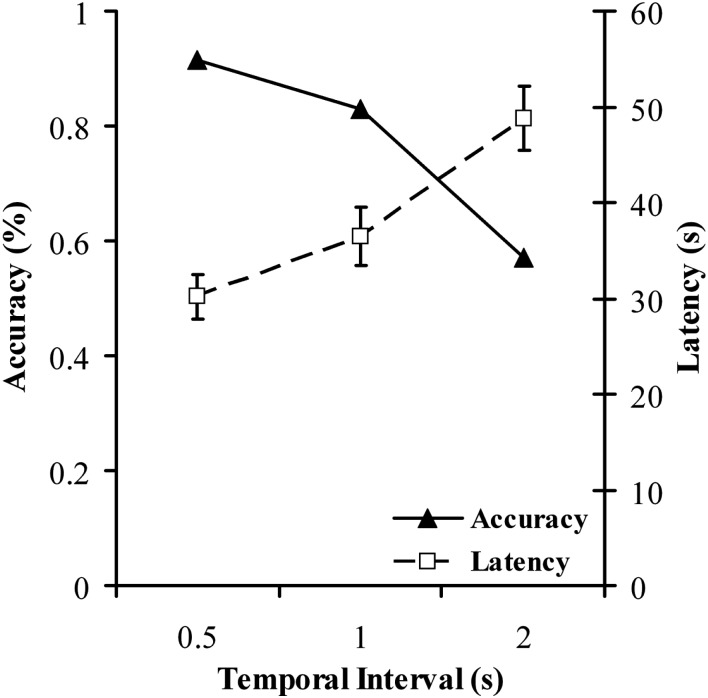
**Mean percentage accuracy and mean response times as a function of temporal delay for the causal attribution task in Experiment 2**. Different symbol and line styles denote accuracy and response time.

Figure 6 shows mean accuracy and response times for each of the three conditions in the causal attribution task. Reaction time was significantly affected by delay, with longer intervals resulting in longer latencies. Binary logistic regression was meanwhile used to assess the impact of delay on accuracy. However, it has previously been reported that a relationship often exists between the speed and the accuracy with which a task is performed or a decision is reached (Garrett, 1922; Schouten and Bekker, 1967; Wickelgren, 1977) commonly referred to as the speed-accuracy tradeoff (SAT). Therefore, as well as being a dependent measure, response time also has the potential to be a determinant of accuracy of choice, and hence was entered into the regression model along with delay. Analysis confirmed that interval length was a significant negative predictor of accurate choice, Wald χ*^2^* = 19.796, β = −1.115, *p* < 0.0005, in other words longer intervals resulted in poorer accuracy. This is consistent with the findings obtained by Young and Nguyen (2009), who also saw accuracy impairments as a consequence of increasing cause-effect intervals, and also with general findings in the literature regarding effects of delay in causal learning (e.g., Shanks et al., 1989). Reaction time was not in this case a significant predictor of accuracy, Wald χ^2^ = 0.104, β = −0.002, *p* = 0.747. Of principle interest for the current paper however was how performance in this experiment correlates with performance in the causal judgment task, delay discounting task, and the BIS. To this end, both mean accuracy and mean response time across the three conditions was calculated.

#### Cross-task comparisons

Neither the delay discounting task (this time) nor the BIS-11 yielded any individual results that can be analyzed in isolation, but instead produced scores for each participant that can be compared to performance in the causal learning tasks. Analysis once again revealed no significant correlation between the causal judgment and delay discounting task, nor was there a correlation between the causal attribution task and the delay discounting task. There was however a significant correlation between participants’ AUC_c_ on the causal judgment task and their mean accuracy on the causal attribution task, *r* = 0.362, *n* = 65, *p* < 0.005. This indicates that participants whose assessments of response-outcome relations were less adversely affected by delay also showed greater ability to identify the correct causal candidate from a number of alternatives even when delays were involved. While many experiments in the literature have confirmed detrimental effects of delay across a wide range of studies, there has been considerably less (if any) research demonstrating that individuals show an internal consistency in the ways in which their causal decisions are affected by delays across learning tasks.

Overall scores on the BIS-11 were not correlated with any of the metrics obtained from the three other tasks, including the delay discounting task, which may be seen as something of a surprise. There was however a marginally significant negative correlation between participants’ scores on the non-planning factor of the BIS-11 and accuracy on the causal attribution task, *r* = −0.229, *n* = 70, *p* = 0.057. This suggests that higher scores on the non-planning second order factor (indicating a lack of planning) tended to result in a lower proportion of accurate choices in the causal attribution task. There were no further significant correlations between the other factors of the BIS-11 (cognitive and motor) and the other three tasks.

### Discussion

#### Summary and interpretation of results

The most notable finding from our second experiment is that there was a strong positive correlation between two very distinct forms of causal learning task. The first was an elemental causal judgment task, where participants were required to evaluate the extent of a putative causal relation by performing responses and observing the subsequent outcomes. The second was a causal attribution task where participants observed three candidates that were all potential causes of a single outcome, and identifying the most likely cause. The two tasks clearly both involve causal thinking, but the disparities between the two are evident, not least in the hypothesis space for each task (see, e.g., Griffiths and Tenenbaum, 2005, 2009). Yet despite their differences, performance in these two tasks was comparable for a given individual, with those doing well on one also tending to do well on the other.

This suggests that when engaging in causal learning, thinking, or reasoning, delays may have a consistent effect upon a given individual from one task to another. It therefore follows that an individual could potentially be categorized as being “delay susceptible” or “delay resistant” depending on their ability to recognize delayed causes or the extent to which they consider delayed effects to be evidence in favor of a causal relation. This is a strong parallel with evidence that has been obtained from studies of intertemporal choice and delay discounting, as reviewed in the Introduction, which suggests that individuals differ in the extent to which they discount delayed rewards. Some individuals (and indeed, cultures, age groups, and other social strata) have a strong preference for immediate rewards and steeply discount delayed rewards. This is often interpreted as an inability to delay gratification and an indication of impulsiveness compared to those who do not discount delayed rewards as steeply and are instead prepared to wait for rewards that are larger in magnitude.

Yet, despite this parallel, and many others earlier outlined in the introduction, we have now twice demonstrated that individual behavior in causal judgment and delay discounting tasks show no correlation. This replication of our earlier finding makes it increasingly likely that the results of this paper constitute evidence of an absence of correlation, rather than merely an absence of evidence. At the same time, the marginally significant correlation between non-planning impulsiveness and accurate causal attribution suggests that causal learning and impulsivity may yet indeed share a connection at some level. However, since the correlation involved the non-planning factor rather than cognitive aspects, then any connection is likely to be based on a lack of forethought or a failure to allow oneself adequate time to make an informed decision, rather than being founded on a common temporal processing mechanism.

On the surface this finding may seem somewhat surprising. The evidence reviewed in the introduction appeared to suggest that because of their inherent similarities, causal judgment and delay discounting may be governed by the same mechanisms. However, one may be even more inclined to suspect that the far more similar processes of delay discounting and probability discounting should share the same cognitive basis. Probability discounting is the process by which the subjective value of a reward declines as the likelihood of its delivery decreases, and therefore would seem to be very tightly connected to delay discounting. Recently, however, there has been an accumulation of data suggesting that a number of variables have different, even opposite, effects on temporal and probability discounting. Green and Myerson (2004) reviewed this evidence and concluded that despite the similarity in the mathematical form of the discounting functions, the patterns of results from their analyses strongly suggest that separate underlying mechanisms are involved for probability and temporal discounting. Hence, with potentially distinct cognitive pathways for these closely allied, perhaps we should not be surprised to find a lack of overlap between individual discounting functions and evaluation of delayed causal relations.

The lack of correlation between the BIS-11 and AUC from the delay discounting task is perhaps surprising, since the latter is often considered to be a behavioral measure of impulsiveness. Indeed numerous studies have previously found a strong positive correlation between BIS-11 and AUC (e.g., McHugh and Wood, 2008), though our study is not the first to show an absence of correlation between the two (Lane et al., 2003; Reynolds et al., 2008). These inconsistencies raise questions over the use of steep temporal discounting as an operational definition of impulsiveness.

#### Implications for theories of learning

Decision making in terms of choice between alternatives involving delays (such as performance on reinforcement schedules) is a direct reflection of the rate at which rewards are devalued by delays. The process of delay discounting however appears to be unconnected to a causal understanding. It would thus seem that this dissociation between discounting and causal learning indicates that simple associative learning cannot form the basis for both these processes. While for the sake of parsimony, a unified learning theory explaining such processes certainly offers appeal, based on the results of this and other studies, such a theory looks set to remain elusive.

To fully explore this conclusion, let us first review some of the essential concepts of learning theory. It is a fairly well-established finding in the behavior analysis literature that animals tend to respond more frequently during variable-interval (VI) reinforcement schedules compared to fixed-interval (FI) schedules (Herrnstein, 1964; Davison, 1969). It has also been demonstrated that animals prefer variable over fixed response-to-reinforcer delays when choosing between two concurrently available alternative response keys (Cicerone, 1976; Bateson and Kacelnik, 1997), thus indicating that the preference for variable reinforcement goes beyond task demands and reflects an inherent property of aperiodicity that makes it preferable. Researchers (McNamara and Houston, 1992; Bateson and Kacelnik, 1995) suggest that such preferences arise from foraging strategies or predatory behavior, which tend to benefit from variability of behavior. However this can also be explained from the perspective of temporal discounting.

If rewards lose their subjective value or associative strength as delays increase, then obviously an early reward contributes more, and a late reward contributes less, relative to a reward occurring at the midpoint of the two. However because of the negatively accelerated shape of the discounting function, the difference between the early and the intermediate reward is greater than the difference between the intermediate and the late reward. In other words, the gain from the early rewards is greater than the loss from the late rewards (compared to an intermediate reward). For an illustrative example, see Greville and Buehner (2010), Figure 1. Thus, a set containing an approximately equivalent number of both early and late rewards will have a greater net subjective value than a set where all the rewards are of intermediate latency. Therefore, assuming that on comparable interval schedules (where the variable schedule will have an even distribution of early and late rewards about the central midpoint of the fixed schedule), although the mean delay-to-reinforcement is approximately equivalent, the variable schedule results in the formation of stronger associations. Applying the temporal discounting principle to animal learning thus provides an account for the apparent preference for variability. The question which then arises, and which is most pertinent to the focal issues being explored in this paper, is whether human causal reasoning follows these same principles.

Greville and Buehner (2010) carried out a series of experiments comparing predictable and variable response-to-reinforcer delays in an instrumental causal judgment task, similar to that employed in the current paper. It was found that conditions where the interval was fixed were routinely judged as more causally effective than those with variable intervals, and furthermore that judgments tended to decline as variability increased. This is in direct opposition to the variability preference observed in animals. Greville and Buehner’s results appear to complement those of the current paper, where we similarly show a dissociation between causal learning with delays and choice involving delayed rewards.

These results would appear to endorse view that causal induction cannot simply be reduced to associative learning. However, this interpretation rests on the assumption that preference for variable reinforcement is a reflection of the associative strength between response and reinforcer, which may not be entirely valid. If instead subjective value and associative strength are dissociable, this may in turn suggest that animals have the capacity to learn associations, or causal connections, without this necessarily resulting in an observable change expressed in behavioral preference. Indeed, a recent variant of associative learning theory, the temporal coding hypothesis (TCH; see, e.g., Miller and Barnet, 1993) posits exactly that. The TCH departs from the traditional associative view by arguing that the temporal relationship between events is encoded as part of the association; that is, the animal learns not only that the US will occur but also when it will occur. This temporal information plays a critical role in determining whether a response is made, and the magnitude and timing of that response. In other words, whether or not an acquired association will be expressed as observable behavior depends on the encoded temporal knowledge (Savastano and Miller, 1998; Arcediano and Miller, 2002). An extension of such an argument would be that an organism may be perfectly capable of recognizing a particular relation, and indeed identifying that relation as stable, but still exercise preference for another schedule that it perceives as perhaps less stable but offering greater potential for reward.

There remain, however, aspects of the design of the current study that could provide alternative explanations for the results obtained. While both the causal judgment task and the delay discounting task are well-established measures in the field, and therefore adequate to assess these respective processes, there are two important distinctions between them. Firstly, the outcome in the causal judgment task had no motivational significance for the participant. Greville and Buehner (2010) suggested that the facilitatory effect of temporal predictability in causal learning found in their studies, which contrasted with the long-established finding of animal preference for variable reinforcement, might in part be attributable to the fact that their causal judgment tasks were appetitively neutral. The same point may be raised here; although the amounts of money in the discounting task were hypothetical, subjects nevertheless tend to respond to such choices as though they were real amounts. In contrast, the outcome in the causal judgment task had no value, hypothetical or otherwise. It is therefore possible that adopting a causal learning task where outcomes provide more tangible reinforcement may have produced different results. However, Shanks and Dickinson (1991) have already contrasted causal judgment with instrumental performance (where participants engaged in a “points-scoring” task) and found that performance and judgment closely mirrored one another. Thus there is little to suggest that such an extension to the current study would yield a different result.

Secondly, while temporal delays in the causal learning tasks are directly experienced, the delay delays in the discounting tasks were merely described. There is considerable evidence that causal judgments made in described situations follow similar patterns to those made based on directly experienced events (Wasserman, 1990; Shanks, 1991; Lovibond, 2003; Greville and Buehner, 2007). We may therefore reasonably conclude that if a described causal learning task had been employed, similar results would have been obtained. However Hertwig et al. (2004) suggest that the same is not necessarily true for choice behavior; for instance, people tend to overestimate and underestimate the probability of rare events in decisions from descriptions and experience, respectively. Future research exploring this question then may seek to see if the same holds true in choosing between immediate and delayed rewards when delays are directly experienced.

#### Neurobiological correlates of associative and causal learning

The evidence from neurological studies reviewed earlier implicated the hippocampus in both the processes of trace conditioning and ability to delay gratification to select a larger delayed reward, and thus formed an important part of the motivation behind the study. We considered that the role of delay in both reducing the subjective value of reward and impairing causal attribution might be similar within individuals, but this proposition was not supported by our experimental findings. The primary implication of these results is that delays impact the two processes in different ways, and thus suggest that local temporal processing, rather than a common temporal processing structure, governs the impact of delays in these processes. We now turn to review further evidence from neurological research that can ballast this argument.

Turner et al. (2004) applied fMRI during a judgment task and found that right lateral prefrontal cortex (PFC) activation is sensitive to the magnitude of prediction error, which is a cornerstone of associative learning models such as the Rescorla-Wagner (Rescorla and Wagner, 1972) model, thus providing direct evidence for a neural basis of the mechanisms proposed in associative learning. However, it is debatable whether the task employed during their experiment constituted a valid test of causal, rather than just simple associative, knowledge. Fugelsang and Dunbar (2005) applied fMRI to a more complex causal judgment task and found that the brain responds differently to incoming data depending on the plausibility of the theory being tested. Specifically, given a plausible causal theory, evaluation of data consistent with that theory recruited neural tissue in the parahippocampal gyrus, whereas evaluating inconsistent data recruited neural tissue in the anterior cingulate, left dorsolateral PFC, and precuneus. Perhaps most relevant to the current article, Satpute et al. (2005) carried out fMRI during a relation-judgment paradigm (see Fenker et al., 2005). Their results revealed that although causal and associative processing shared several regions of activity in common, accessing causal knowledge produced patterns of activity in left dorsolateral prefrontal cortex (DLPFC) and right precuneus that were absent during associative judgment, even after correcting for task difficulty. They concluded that evaluating causal relations involves additional neural mechanisms relative to those required to evaluate associative relations. Indeed, further dissociations can be made at the neurological level even between basic associative processes. Myers and Davis (2002) report that acquisition and extinction appear to be governed by fundamentally different neural mechanisms in different learning paradigms, evident particularly when comparing extinction of fear conditioning and conditioned taste aversion. Each appears to recruit its own configuration of cellular mechanisms, perhaps as a function of task difficulty or the nature of the CR, CS, or US.

These findings appear to support a distinction between causal and associative learning. While there are certainly elements in common between the two processes, both in their neurological bases and at a cognitive or computational level, a number of important contrasts remain. Specifically, it appears that causal learning involves an additional layer of complexity, and recruits additional neurological structures, than associative learning.

#### Critique and future directions

This study is the first, as far as we are aware, to experimentally explore a potential link between causal learning and discounting. As such, the work was largely exploratory in nature and the methodology untested, so it is entirely possible that superior methods of comparing discounting functions with causal judgment data may be constructed. While the AUC has become a standard measure for temporal discounting, no such universally accepted measure exists for the causal judgment task. Our application of this procedure to our causal judgment task may thus be open to some criticism. For instance, the delays in the two tasks differ greatly in duration; seconds for the causal judgment task and months and years for the discounting task; moreover, as mentioned earlier, delays are experienced in the former while described and imagined in the latter. In addition, we only used three delays in the causal judgment task, rather than the seven in the delay discounting task; studying judgments over a much broader range of delays may provide a more finely tuned measure of the causal judgment data.

An alternative suggestion might be to contrast fixed and variable delays, as in Greville and Buehner’s (2010) studies, and investigate whether preference for fixed vs. variable relations has any connection to temporal discounting. Despite the general trend of preference for predictability shown in Greville and Buehner’s studies, individual participants may deviate from this trend and exhibit preference for variability. It would be interesting to see if such a preference arises from a contiguity bias, whereby the potential for immediate reinforcement in variable conditions overrides the impression of stability provided by fixed conditions, and whether such a contiguity bias is correlated with steep temporal discounting and impulsivity.

Finally, steep discounting has often been linked in the literature with impulsivity (Richards et al., 1999), which in turn has been linked to a number of socially maladaptive behaviors, including violence, drug abuse, and pathological gambling (Steel and Blaszczynski, 1998; Fishbein, 2000). As a result, considerable effort has been devoted to the development of potential interventions for impulsive behavior, and the current line of research may help provide further insights. For instance, although overall we found little evidence of any relationship between impulsivity and causal understanding, there was a marginally significant negative relationship between non-planning impulsiveness and correct identification of causes. An adaptation of this paradigm into a training game designed to improve causal attribution might therefore be a potential means of reducing the propensity for non-planning impulsivity. Though the current study does not yet provide strong enough evidence that could inform clinical practice, future research might suggest avenues for the development of new therapeutic strategies.

### Conclusion

To summarize, this study represents an early step in exploring the potential relationship between areas of learning that have previously tended to be somewhat shielded from one another in the literature. Our results indicate that delays have a consistent influence in tasks involving causal learning, and that a given individual may be affected in the same way by delays across different causal learning tasks. However no such correlation exists between the manner in which delays hamper causal learning and the rate at which the subjective value of delayed rewards is discounted. Taken together with the results of Greville and Buehner (2010) and those of Satpute et al. (2005), the implication is that there is a dissociation between reinforcement learning and causal inference, and the effects of time in these learning processes cannot be attributed to a common temporal processing mechanism. The results have valid implications for current theories of learning as well as considerations for interventions in problem behavior and psychotherapy. It is our hope that further research will shed more light on this topic and more precisely identify those facets of learning processes that are idiosyncratic and those that form common elements between processes.

## Conflict of Interest Statement

The authors declare that the research was conducted in the absence of any commercial or financial relationships that could be construed as a potential conflict of interest.

## Footnotes

^1^It is worth mentioning here that while trace conditioning involves a delay (trace interval) separating CS and US, counterintuitively, delay conditioning does not; CS either follows immediately or co-terminates with US. The “delay” in the term refers to that between CS onset and US onset.
